# T Helper Cell Lineage-Defining Transcription Factors: Potent Targets for Specific GVHD Therapy?

**DOI:** 10.3389/fimmu.2021.806529

**Published:** 2022-01-05

**Authors:** Julia Campe, Evelyn Ullrich

**Affiliations:** ^1^ Experimental Immunology, Children’s University Hospital, Goethe University Frankfurt, Frankfurt am Main, Germany; ^2^ Children’s University Hospital, Goethe University Frankfurt, Frankfurt am Main, Germany; ^3^ Frankfurt Cancer Institute, Goethe University Frankfurt, Frankfurt am Main, Germany; ^4^ German Cancer Consortium (Deutsches Konsortium für Translationale Krebsforschung (DKTK)), Partner Site Frankfurt/Mainz, Frankfurt am Main, Germany

**Keywords:** aGVHD, GvL, CD4+ T cells, T helper cell differentiation, transcription factors

## Abstract

Allogenic hematopoietic stem cell transplantation (allo-HSCT) represents a potent and potentially curative treatment for many hematopoietic malignancies and hematologic disorders in adults and children. The donor-derived immunity, elicited by the stem cell transplant, can prevent disease relapse but is also responsible for the induction of graft-versus-host disease (GVHD). The pathophysiology of acute GVHD is not completely understood yet. In general, acute GVHD is driven by the inflammatory and cytotoxic effect of alloreactive donor T cells. Since several experimental approaches indicate that CD4 T cells play an important role in initiation and progression of acute GVHD, the contribution of the different CD4 T helper (Th) cell subtypes in the pathomechanism and regulation of the disease is a central point of current research. Th lineages derive from naïve CD4 T cell progenitors and lineage commitment is initiated by the surrounding cytokine milieu and subsequent changes in the transcription factor (TF) profile. Each T cell subtype has its own effector characteristics, immunologic function, and lineage specific cytokine profile, leading to the association with different immune responses and diseases. Acute GVHD is thought to be mainly driven by the Th1/Th17 axis, whereas Treg cells are attributed to attenuate GVHD effects. As the differentiation of each Th subset highly depends on the specific composition of activating and repressing TFs, these present a potent target to alter the Th cell landscape towards a GVHD-ameliorating direction, e.g. by inhibiting Th1 and Th17 differentiation. The finding, that targeting of Th1 and Th17 differentiation appears more effective for GVHD-prevention than a strategy to inhibit Th1 and Th17 cytokines supports this concept. In this review, we shed light on the current advances of potent TF inhibitors to alter Th cell differentiation and consecutively attenuate GVHD. We will focus especially on preclinical studies and outcomes of TF inhibition in murine GVHD models. Finally, we will point out the possible impact of a Th cell subset-specific immune modulation in context of GVHD.

## Introduction

Allogenic hematopoietic stem cell transplantation (allo-HSCT) represents a potent and potentially curative treatment for many hematopoietic malignancies and hematologic disorders in adults and children. Its success is based on a complete replacement of the patients’ immune system by a myeloablative conditioning regimen and reconstitution from a healthy donor stem cell graft. The donor derived immunity can prevent disease relapse but is also responsible for the main complication of allo-HSCT, the graft-versus-host disease (GVHD).

Acute GVHD pathophysiology is not completely understood yet. In general, acute GVHD is driven by the inflammatory effect of donor T cells upon antigen-recognition of allo-antigens presented by host antigen-presenting cells (APCs). The subsequent alloreactive cytotoxicity of activated T cells effects the GVHD target organs (gastrointestinal tract, skin, and liver) and leads to an amplification loop of inflammation there.

Since several experimental approaches indicate that CD4 T cells play a key role in initiation and progression of acute GVHD, the contribution of the different CD4 T helper (Th) cell subtypes in the pathomechanism and regulation of the disease is a central point of current research. Acute GVHD is thought to be driven by a Th1/Th17/Th22 axis whereas Treg cells are attributed to attenuate GVHD effects. As the differentiation of each Th subset highly depends on the specific composition of activating and repressing transcription factors (TFs), these present a potent target to alter the Th cell landscape towards a GVHD-ameliorating direction by the inhibition of Th1 and Th17 differentiation. In this review, we discuss the current advances of potent of potent TF inhibitors in order to alter Th cell differentiation and attenuate GVHD in murine models.

## T Helper Cell Subsets and Differentiation

**Figure 1:**
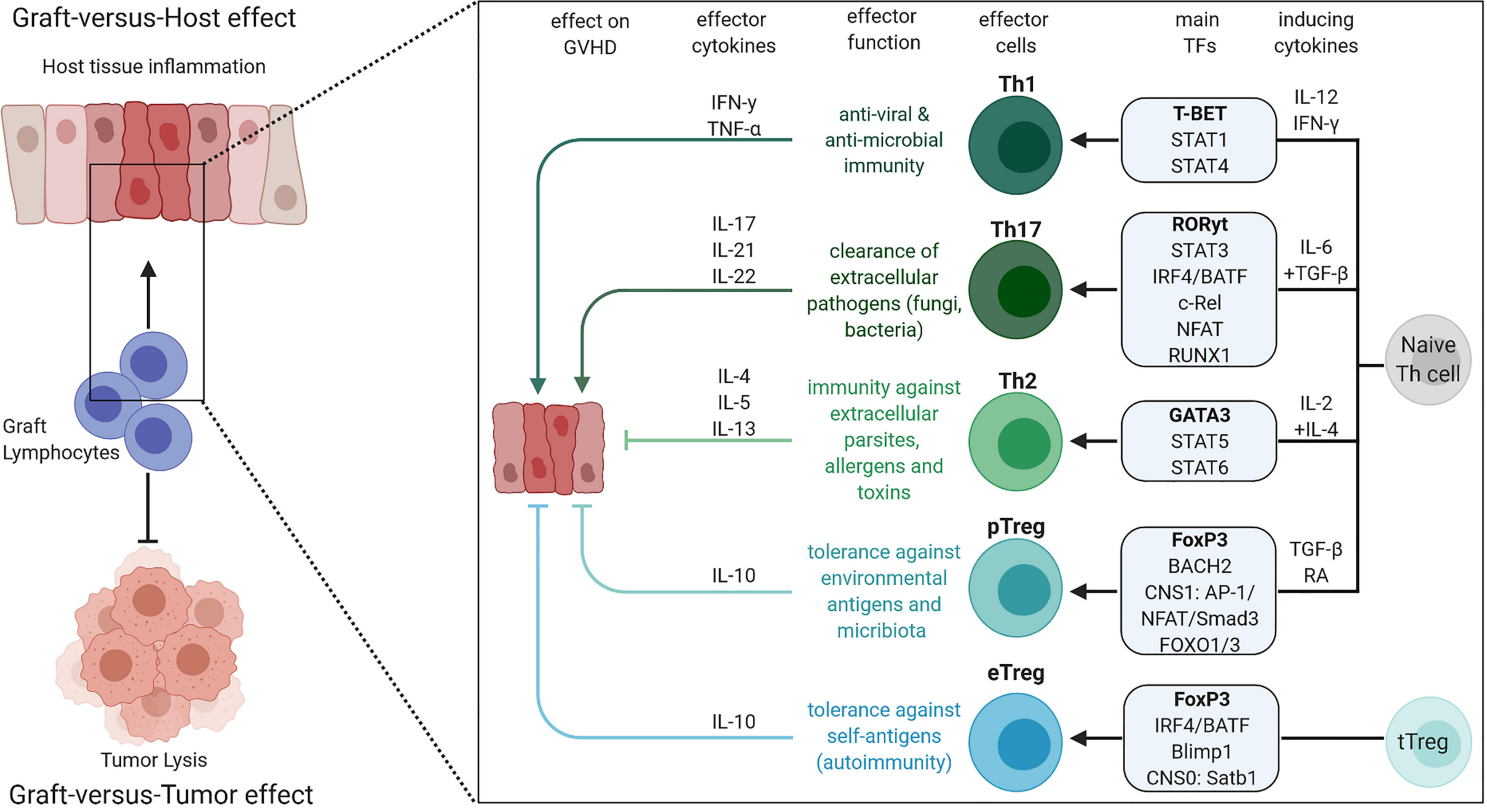
Overview of Th1, Th2, Th17, eTreg and pTreg differentiation, their effector cytokines, roles in the immune system and impact on GVHD. The figure was created with BioRender.com.

To date, eight different T helper cell types are known: Th1, Th2, Th9, Th10, Th17, Th22, follicular T helper cells (Tfh) and regulatory T cells (Treg). Th cell lineages derive from naïve CD4 T cell progenitors and lineage commitment is initiated by the surrounding cytokine milieu and subsequent changes in the TF profile. Each T cell subtype has its own effector characteristics, immunologic function, and lineage specific cytokine profile, leading to the association with different immune responses (**Figure 1**). In this review we will focus on the Th1, Th2, Th17 and Treg subsets, the involved TFs in their differentiation as well as their impact on GVHD.

### Th1 and Th2 Cells

In 1986, Mosmann and colleagues identified two distinct classes of CD4 helper T cells, which exhibited a different cytokine profile. The differentiation in these two classes, later called Th1 and Th2, was found to be stable and deterministic (1). Th1 cells differentiate in the presence of interferon (IFN)-γ and interleukin (IL)-12 to produce their effector cytokine IFN-γ, which has high relevance for anti-viral and anti-microbial immunity (1–3). In contrast, Th2 cells differentiate in the presence of IL-2 and IL-4 and produce the effector cytokines IL-4, IL-5 and IL-13, which play an important role in the immune response against extracellular parasites, bacteria, allergens, and toxins (1, 4–7).

In the early 2000s, Szabo et al. discovered that the underlying mechanisms of the Th1/Th2 paradigm was the initiation or repression of distinct genetic programs upon activation, directed by Th lineage specific master transcription factors (8). With this regard, T-bet was described as a master regulator of Th1 cells, which induces IFN-γ production by activating Th1 genetic programs while repressing Th2 responses (8–12). A few years earlier, GATA3 was characterized as a master transcriptional regulator for Th2 cell differentiation (13, 14).

Further studies on the mechanisms, how T-bet and GATA3 mediate Th1 and Th2 differentiation respectively, revealed the mutual inhibition of the two master TFs and the involvement of many more interacting molecules and relevant signaling cascades (15–17). T−bet was found to be induced by Signal Transducers and Activators of Transcription Protein 1 (STAT1) and IFN-γ during T cell activation and to induce STAT1 dependent processes as the induction of Interleukin-12 receptor subunit beta-2 (IL-12Rβ2) (12). Additionally, STAT4 which is activated by IL-12, and the downstream acting TF c-Rel were identified as crucial transcriptional regulators for Th1 differentiation (15, 18–22). In contrast, Th2 differentiation was associated with IL-2 dependent STAT5 signaling and IL-4 dependent STAT6 signaling pathways, which induce the expression of GATA3, IL-2 receptor (R)a and IL-4Ra as well as IL-2 and IL-4 effector cytokines (23–25).

### Th17 Cells

Th17 cells were first described as an independent and distinct Th subset from Th1 and Th2 cells, producing IL-17a, IL-17f, IL-22 and IL-21 as effector cytokines in the early 2000s (26, 27). First thought that IL-23 was the inducing cytokine for Th17 cells, three groups simultaneously discovered that TGF-β and IL-6 induced Th17 differentiation (28–30), while IL-1β and tumor necrosis factor (TNF)-α can potentiate Th17 differentiation in presence of IL-6 and transforming growth factor beta TGF-β (31–33). The leading role of Th17 cells is the clearance of extracellular pathogens as fungi and bacteria but dysregulation of Th17 effects is associated with various autoimmune diseases like inflammatory bowel disease, rheumatic arthritis, experimental autoimmune encephalomyelitis (EAE), and multiple sclerosis [reviewed by Tesmer at al. (34)].

In 2006, the transcription factor retinoid acid-related orphan receptor (ROR)γt (*Rorc*) was identified to be uniquely expressed in mouse Th17 cells and necessary for Th17 differentiation (35). Besides, RORγt as master transcription factor, several other TFs were described to be crucial for Th17 differentiation and function. STAT3 was found to drive the transcription of Th17 specific genes like *Il17a, Il17f and Il23r* (36, 37) and to suppress TGF−β-induced forkhead box protein 3 (FoxP3) expression and hence regulatory T cell differentiation (28). Interferon Regulatory Factor 4 (IRF4) and Basic Leucin Zipper ATF-Like Transcription Factor (BATF) also play a significant role in Th17 differentiation by initiating the transcription of Th17 defining genes as *Il17, Il21, Il23r* and *Rorc* (38–40). IRF4 was also shown to physically interact with RORγt (38) and STAT3 (36). The transcriptional regulators c-Rel, p65, nuclear factor of activated T cells (NFAT)c2 and Runt-related transcription factor 1 (RUNX1) were found to directly regulate RORγt by binding to the *Rorc* promotor (41–43). Additionally, RUNX1 and hypoxia-inducible factor 1-alpha (HIF1α) physically interact with RORγt to potentiate or co-activate IL-17a expression (44, 45). Importantly, T-bet and GATA3 can inhibit RUNX1 expression or binding to DNA respectively which inhibits Th17 differentiation.

### Regulatory T Cells

In contrast to the immune effector function of Th1, Th2 and Th17 cells, regulatory T cells (Tregs) are characterized by their immunosuppressive capacity and are essential mediators of self-tolerance. Already in the 1960’s it was found that a thymus-derived cell population was mediating immunologic tolerance. Later on, Sakaguchi and colleagues characterized these cells further as CD4 T cells expressing the IL-2 receptor alpha chain (CD25) (46). However, it was unclear if Tregs represent a distinct cell line until the Treg master transcription factor FoxP3 was discovered (47, 48). The importance of FoxP3 for Treg differentiation is well displayed by scurfy mice which lack FoxP3 expression and suffer from inflammatory autoimmune syndrome (47, 49). Additionally, the maintenance of FoxP3 expression after differentiation is essential for Treg immunosuppressive function (50, 51). Besides the expression of FoxP3, the development, maintenance, and function of Tregs also highly depends on TGF-β (52–55).

In contrast to other effector T helper cells, regulatory T cells differentiate in the thymus [thymus-derived Tregs (tTregs)], dependent on high affinity interaction with complexes of MHC-II and tissue-restricted self-antigens and IL-2 receptor signaling (56). However, Tregs can also differentiate from naïve T cells in the periphery (pTregs), sometimes also referred to as induced Tregs (iTregs). These cells are induced by non-self-antigens and are most likely mediating immunologic tolerance of environmental antigens and commensal microbiota [reviewed by Lee et al. (57)].

pTreg and tTreg differentiation are implemented on a transcriptional level by different involvement of regulatory elements, four conserved non-coding sequences (CNSs) of the *Foxp3* locus (58). CNS1, regulated by the transcription factors Activator protein 1 (AP-1), NFAT, Small mothers against decapentaplegic homolog 3 (Smad3) and Forkhead box O (FOXO) (57, 59–62), was found to be necessary for pTreg but not for tTreg development, while CNS0, regulated by special AT-rich sequence-binding protein-1 (Satb1) is essential for tTreg generation (63). CNS2, which is regulated by the TF Protein C-ets-1 (Ets-1), cAMP response element-binding protein (CREB), RUNX, STAT5, NFAT and c-Rel is important for stable FoxP3 expression during differentiation and functionality of Tregs (58, 64–69). In contrast, CNS3 which is regulated by c-Rel and FOXO TFs influences Treg cell numbers (57, 58, 62). Additionally, gaining the full suppressor function of tTregs as effector Tregs (eTregs) depends on the transcription factors IRF4 and B lymphocyte-induced maturation protein-1 (Blimp-1), which drive the expression of the immunosuppressive cytokine IL-10 (70), while BACH2, a transcriptional repressor, inhibits the genomic binding of IRF4, and mediates pTreg differentiation and maintenance (71).

### Cross Regulation of T Helper Cell Differentiation

In general, Th differentiation fates are tightly connected and regulated. For example, Th1 and Th2 cells inhibit the development of each other by their lineage specific transcription factors (72, 73) and by the cytokines IFN-γ and IL-4 (74, 75). The differentiation of Th17 cells can also be inhibited by these cytokines and by the expression of the TF T-bet (26, 72, 76). However, fully differentiated Th17 cells are resistant to IFN-γ and IL-4 inhibiting effects *in vitro* (27).

The T cell fate of Th17 and Tregs is connected especially tightly, as many factors were shown to have a reciprocal role in Th17 and Treg development. One reason for that is the response of both cell types to TGF-β signaling. However, IL-6 regulates the TGF-β response between both subsets, since it is necessary for Th17 induction, while it inhibits TGF-β induced Treg differentiation (77, 78). On the contrary, Tregs can lose their FoxP3 expression and reprogram to IL-17 secreting cells in the absence of TGF-β (79). Many more regulatory pathways also show that contradictive role in Th17 and Treg development. The activation of mammalian target of rapamycin (mTOR) *via* HIF1α promotes Th17 differentiation, whereas the lack of HIF1α and mTOR drives Treg development (80). As another example, inhibiting protein kinase CK2 was shown to block Th17 development and promotes Treg cell differentiation in mice due to a defect in STAT3 phosphorylation (81). FoxP3 itself, can also associate with RORγt and inhibit RORγt activity (82). GATA3 was shown to play a vital role in Treg differentiation as it binds to CNS2 elements and represses the development of a Th17 phenotype (83). A similar effect was reported on IL-2 which promotes Treg development and inhibits Th17 differentiation dependent on STAT5 (84). In general the opposing regulation of genes like *Il17* through STAT3 and STAT5 seems to be a crucial mediator of reciprocal Th17/Treg differentiation (85).

## The Impact of Th Cells in GVHD

The role of different Th-subsets in GVHD-induction and progression has been investigated with various approaches and GVHD-mouse models for quite a long time. First focusing on Th-subset specific cytokines, these studies mostly provided paradoxical results regarding the role of Th1, Th2 and Th17 cells in GVHD. However, following experiments with Th-defining TF knockout T cells improved the understanding of Th-subset involvement in GVHD. Overall, Th2 and Tregs are subsets with a protective effect on GVHD while Th1 and Th17 cells promote GVHD induction and progression. The following paragraph will give more detailed information on the various approaches revealing the role of the different Th subsets in aGVHD.

### Protective T Helper Cell Subsets in GVHD

First studies examined the effect of Th2-associated cytokines in GVHD in the 1990’s. Injection of the Th2 inducing cytokines IL-2 and IL-4 led to Th2 polarization and protected recipient mice from GVHD-associated mortality (86, 87). Comparable results were observed after the administration of Granulocyte-macrophage colony-stimulating factor (GM-CSF) to recipient mice, which induced IL-4 production and inhibited GVHD-development (88). Another study confirmed the GVHD-attenuating effect of IL-4 produced by Th2 cells, also having a skewing effect on Th2 cytokines (89). On the contrary, other studies showed that the absence or neutralization of IL-4 ameliorated GVHD, implying a detrimental role of Th2 cells (90, 91). However, these contradicting results regarding the role of IL-4 in GVHD may be based on different mouse models and experimental settings (92). Despite the overall protective role of IL-4 secreting Th2 cells in GVHD, the location of these cells might define their pathogenic relevance, as they were associated with pathophysiological changes in the lung, but not in colon, liver, and skin during GVHD (93). IL-13, another Th2 effector cytokine, was also shown to have an ameliorating effect on GVHD. Although one study correlated IL-13 levels with GVHD severity in patients (94), transplantation experiments of IL-13^-/-^ cells in an established mouse GVHD model resulted in increased mortality and decreased Th2 cytokine levels but elevated serum levels of TNF-α, a critical mediator of GVHD, in these mice (95). Further studies showed the counteracting role of IL-13 to TNF-α production and its augmenting role in IL-4 and IL-5 secretion following allo-bone marrow transplantation (96), supporting the notion that IL-13 has a protective function in GVHD. In general, the transplantation of Th2 cells to recipient mice showed beneficial effects on GVHD-survival (97, 98) and an alteration of the Th1/Th2 balance towards the Th2 cells leading to increased IL-4 levels and attenuated GVHD (98–100). Ultimately, a study investigating IL-4, IL-5, IL-9, and IL-13 quadruple cytokine-deficient T cells in a well-established mouse model demonstrated that combined Th2 cytokine deficiency resulted in enhanced T cell proliferation, higher TNF-α, IL-2, IFN-γ and IL-17a serum levels and overall aggravated GVHD (101).

A few further experiments on Th2 defining TFs gained similar results in GVHD models. Atorvastatin (AT) treatment was shown to modulate Th1/Th2 differentiation by inhibiting the production of the isoprenoid derivates farnesly-pyrophosphate (PP) and geranylgranyl-PP, of the mevalonate pathway. Inhibition of these isoprenoid derivates combined by AT or individually by a farnesyltransferase inhibitor (FTI) or a geranylgeranyltransferase inhibitor (GGTI) respectively, resulted in an upregulation of GATA3, and in case of AT and FTI treatment also an downregulation of T-bet expression in antigen-primed T cells (102). GGTI and FTIs were also shown to have ameliorating CD4 T cell specific effects on GVHD while sparing CD8 T cells in their capacity to mediate GVL and protect from viral infections (103). AT treatment also induced Th2 polarization and cytokine secretion and inhibited GVHD development by partially acting through STAT6, a transcription factor essential for Th2 differentiation in response to IL-4 and IL-13 (23, 104, 105). STAT6 was shown to be required for Th2 involved NKT-cell mediated GVHD prophylaxis (106). Additionally, transplanted STAT6^-/-^ T cells, unable to differentiate to Th2 cells skewed towards Th1 cells and mediated aGVHD with major involvement of the colon. On the contrary, STAT4^-/-^ T cells, which predominantly differentiated to Th2 cells, showed less severe signs of GVHD but later involvement in skin pathology (107). STAT5, another critical TF in Th2 differentiation, was found to have a dual role in Th2 and Treg differentiation in GVHD, as overexpression of STAT5 led to increased Treg numbers and attenuated GVHD, while in the absence of Tregs, anti-inflammatory Th2-cytokines increased (108).

Tregs are the second CD4 T cell subset which play a protective role in GVHD. In general, responsible for immune homeostasis and balanced immune responses, Tregs have an outstanding role in controlling GVHD development. First experiments on CD4+ CD25+ Tregs in GVHD showed that depletion of these cells aggravated GVHD, while supplementation with Tregs had the contrary effect (109, 110). The capacity of Tregs to attenuate GVHD was associated with their expansion-inhibiting effect on allogeneic T cells in the early phase of GVHD (111). The beneficial effect of Tregs in GVHD prevention was demonstrated in fully allogeneic, haploidentical and xenograft mouse models (111–116). FoxP3 expression was additionally found to negatively correlate with GVHD severity in patients (117). Importantly, murine, and human Tregs attenuate allogeneic T cell reactions, without impeding the graft-versus-leukemia (GVL) effect (111, 118–121). The use of *in vitro* induced Tregs (iTregs) as a GVHD therapeutic revealed effective protection in the early phase after transplantation but unstable FoxP3 expression over time led to aggravation of GVHD, making this approach less promising as initially thought (122, 123). However, additional combinatory induction with IL-2 and rapamycin was shown to stabilize FoxP3 expression in these cells (113, 124), which enabled the first successful application of iTregs as GVHD-prophylactic therapy to humans (125).

Despite this broad outline of Treg research in GVHD, many recent publications have already summarized the role of Tregs in GVHD in a detailed way (126–128), for which reason we will not go into further details at this point.

Overall, Th2 and Tregs were shown to have an attenuating and protective role in GVHD. While Th2 cells can still mediate local GVHD-associated pathophysiological changes in the lung, Tregs are an overall protective cell population in GVHD having crucial homeostatic functions, which are tightly regulated in balance with other Th-subsets.

### Detrimental Th Subsets in GVHD

Contradicting first studies on Th1 cytokines in the 1990’s led to unconclusive results regarding the role of Th1 cells in GVHD. The main Th1-inducing and -associated cytokines IL-2, IFN-γ and IL-12 were found to ameliorate GVHD in several early studies which indicated a protective function of Th1 in GVHD (129–132). However, other groups showed, that increased IFN-γ levels in serum correlated with GVHD severity (133, 134) and that IFN-γ was critical for tissue pathology during GVHD (97). Besides the beneficial role of IFN-γ in the induction of GVHD-associated effects in the lung (135), it was shown to have adverse effect in acute GVHD pathology in the GI tract (93, 136–138). Additionally, the effect of IFN-γ in GVHD was found to be dependent on the irradiation regimen used (139). Overall, the reciprocal effect of IFN-γ in GVHD seems to be highly dependent on conditioning, location, timing, and the stage of allo-immune response [reviewed by Lu and Waller, (140)].

Similar to IFN-γ, contradicting findings were made, when Th17-associated cytokines were assessed in GVHD mouse models. One study suggested a protective role of IL-17a in GVHD, as IL-17^-/-^ T cells accelerated GVHD while the systemic administration of IL-17a and the neutralization of IFN-γ prevented this effect (141). Other studies reported improved transplantation outcomes when IL-17a^-/-^ T cells were used (142) and severe GVHD induction when *in vitro* generated IL-17+ cells were infused (143). Altogether, these studies indicated that, similar to IFN-γ, the role of IL-17 in GVHD is dependent on timing and conditioning regimen. IL-17 probably contributes to early development of GVHD but is dispensable for overall GVHD induction (142). Neutralization of the IL-17 inducing cytokine TGF-β was shown to increase aGVHD severity indicating an ameliorating effect of Th17 cells in GVHD (144). However, TGF-β is also relevant for the differentiation of Tregs which are GVHD protective, and its absence resulted in enhanced Th1 cell proliferation indicating Th17-independent mechanisms that lead to enhanced GVHD (144). IL-6, which induces TGF-β dependent differentiation of Th17 but not Treg cells, was found to play a relevant role in GVHD induction, as blocking of the IL-6R led to reduced GVHD pathology and Th1/Th17 cells in GVHD target organs, while absolute numbers of Tregs increased (145). However, another study showed that short-term administration of IL-6 could not confirm these beneficial effects (146). Differences between the design of these two studies indicate that the effect of IL-6 on GVHD development is dependent on conditioning, the used model, and the duration of therapy.

TNF-α, another Th1-associated cytokine, which also promotes Th17 differentiation, was shown to drive GVHD pathophysiology on several stages. For example, TNF-α is responsible for early intestinal GVHD-related toxicity (147) and TNF-receptor 1 (TNFR1) levels strongly correlate with GVHD severity (148). Additionally, the attenuating effect of TNF-blocking therapy in GVHD underlines the detrimental role of TNF-α in GVHD (149). Similarly, inhibiting the Th17 effector cytokines IL-21 and IL-23 decreased GVHD severity in various mouse models (150–152).

However, cytokines can derive from different cell types and do not necessarily represent the involvement of respective Th cell subsets. Hence, experiments examining subset defining TF knock-out CD4 T cells shed more light on the relevance of different Th cell subsets in GVHD and identified Th1 and Th17 cells as the relevant subsets promoting GVHD.

First TF-knock-out experiments to investigate the influence of Th1 differentiation on GVHD were performed with STAT6^-/-^ and STAT4^-/-^ T cells. STAT6^-/-^ T cells are unable to differentiate to Th2 cells but instead show enhanced Th1 responses (23, 104, 153). In contrast, lack of STAT4 in T cells leads to impaired Th1 differentiation (154). Nikolic and colleagues investigated STAT6^-/-^ and STAT4^-/-^ T cells in a GVHD mouse model and found that STAT6^-/-^ T cell recipients showed an earlier and more severe course of GVHD with severe inflammation in the GI tract in comparison to STAT4^-/-^ T cell recipients, while only the latter group displayed severe skin disorders (107). These results indicate the detrimental role of Th1 cells in GVHD mainly affecting the GI tract but not liver and skin. Recipients of T cells with STAT1 KO, another critical STAT TF for Th1 development, also resulted in the attenuation of GVHD and increased Treg expansion (155). Comparable results were obtained in GVHD experiments with c-Rel KO T cells, which showed a dramatically reduced ability to induce GVHD in various mouse models, defects in Th1 and Th17 differentiation, enhanced Treg differentiation and a preserved Graft-versus-leukemia (GvL) effect (156).

Experiments with T cells deficient for T-bet and RORγt, the master TFs of Th1 and Th17 cells respectively also confirmed that these subsets are the most involved Th cells in GVHD induction and development. Transplanted T-bet deficient T cells screwed to Th2, Th17 and Treg subsets and led to attenuated GVHD, especially in the gut (157). The absence of RORγt in T cells only had little impact and RORγt was dispensable to induce GVHD development in two independent studies (157, 158), while one study reported an attenuated effect on GVHD if both isoforms, RORγ and RORγt were absent in CD4 transplanted T cells due to KO of the entire *Rorc* locus (159). However, T-bet and RORγt double KO T cells, which showed a defective differentiation of Th1 and Th17, and increased Th2 and Treg cells, induced less GVHD than T-bet KO T cells alone. This finding suggests a synergistic effect of RORγt-induced Th17 cells on Th1-mediated GVHD induction (157).

In addition, TFs linked to the reciprocal differentiation to Th17 versus Treg cells were also found to play a crucial role in attenuation of GVHD. For example, recipients of T cells with a STAT3 deficiency, a TF crucial for Th17 development, showed attenuated GVHD development and increased numbers of pTregs (160).

Summarized, Th1 and Th17 cells synergistically are the main Th subsets driving GVHD, especially with detrimental pathological effects on the GI tract. Blocking of Th1 and Th17- transcription factors was found to be a more effective strategy to prevent GVHD, than blocking Th1 and Th17-involved cytokines. Hence, the use of specific TF-blocking agents is a promising strategy to treat GVHD in the future. The following paragraph will give deeper insights in recent literature reporting the effect of a variety of Th-subset specific TF blocking agents in murine GVHD models.

## Potential Strategies to Target Transcription Factors of T Helper Cell Development in GVHD

As described earlier, experiment with various Th-differentiation associated TF knock-out T cells revealed efficient attenuation of GVHD in different transplantation models. Hence, inhibition of these TFs by target-specific inhibitory agents offers a potent strategy for GVHD prophylaxis and therapy.

Several commonly used GVHD therapeutics also rely on the modulation of TF expression or activity. Calcineurin inhibitors (CNIs) like Cyclosporine A (CyA) of tacrolimus (FK506) for example block TCR-proximal signaling by inhibition of NFAT. Even though CNIs remain standard of care for GVHD prevention, they also interfere with the valuable GVL-effect by impairing donor immunity and disruption of Treg function and survival (161–165). Combinatorial therapy with mTor inhibitors like Rapamycin (Sirolimus) and/or low-dose IL-2 administration have already shown to improve Treg reconstitution after allo-hematopoietic cell transplantation (164, 166–170).

The following section will provide more detailed information on various new therapeutic agents, divided by substance classes, which have been successfully evaluated in GVHD alone or in combination with standard of care therapeutics in the recent years (**Tables 1**, **2**). Importantly, if not indicated by the respective clinical trial number or mentioned explicitly, this paragraph mostly summarizes results from pre-clinical GVHD mouse models and not from studies in patients. Most of them rely on the strategy of targeting TFs that mediate the reciprocal effect between Th17/Th1 and Treg differentiation, hence inducing a homeostatic effect by skewing CD4 T cell differentiation towards Tregs while preserving the GVL effect.

**Table 1:** Summary of pre-clinical studies on Th-differentiation targeting TF inhibitors.

Class	Sub-class	Compound	Target	murine aGVHD model	Effect GVHD	Effect GVL	Effect Th differentiation	Reference
Epigenetic regulators	HDACi (short-chain fatty acid)	Valproic acid (VPA)	AKT	MHC mismatch model: BL/6→BALB/c	ameliorated	preserved	Th1 ↓ Th17 ↓	(171)
	HDACi(sirtuin inhibitor)	Ex-527	Sirt-1	MHC mismatch model:BL/6→BALB/c	ameliorated	preserved	Th1 ↓ Th17↓ Tregs ↑	(172)
	HDACi(hydroxamic acid)	Vorinostat (SAHA)	STAT3/STAT1	MHC mismatch model:BL/6→BALB/c	ameliorated	**-**	**-**	(173)
	HDACi(cyclic peptides)	Romidepsin	STAT3/STAT1	MLR	ameliorated	**-**	**-**	(174, 175)
Kinase inhibitors	JAK/STAT Inhibitors	Ruxolitinib (INCB018424)	JAK1/2	MHC mismatch model:B6→BALB/c	ameliorated	preserved	Th1 ↓ Th17↓ Tregs↑	(176, 177)
	JAK/STAT Inhibitors	Itacitinib (INCB039110)	JAK1	MHC mismatch model:B6→BALB/c; xenogeneic model	ameliorated	preserved	Tregs **↑**	(178–180)
	JAK/STAT Inhibitors	Pacritinib	JAK2	minor histocompatibility antigen-mismatched model BALB/b→BALB/c;MLR (human);human skin graft rejection model	ameliorated	preserved	Th1↓ Th17↓ Th2↑	(181)
	JAK/STAT Inhibitors	Pacritinib +S3I-201 +Rapamycin (Sirolismus)	JAK2+STAT3+mTOR	xenograft model	ameliorated	preserved	Th1 ↓only PAC/SIR or S3I/SIR:Th17↓ Tregs↑	(166)
	JAK/STAT Inhibitors	Fedratinib (TG101348)	JAK2/STAT3 axis	MLR	ameliorated	**-**	Th1↓ Th17↓ Tregs↑	(182)
	JAK/STAT Inhibitors	Tofacitinib (CP-690550)	JAK3	semiallogeneic MHCII-disparate model B6→(B6xbm12)F1;MLR	ameliorated	**-**	Th1↓	(183)
	ROCK1/2 Inhibitors	Fausidil	Rho kinase (ROCK1 and ROCK2)	semiallogeneic MHC-disparate modelC3H→ (B6C3)F1	ameliorated	–	–	(184)
	ROCK1/2 Inhibitors	Belomosudil (KD025)	ROCK2	major MHC mismatch model of multiorgan cGVHD; minor MHC mismatch model of sclerodermous GVHD	ameliorated	–	Tfh ↓ Tfregs↑	(185)
	other Inhibitors	ONO-7790500	ITK	semiallogeneic MHC-disparate modelB6→ (B6D2)F1	ameliorated/delayed	preserved	Th1 ↓Th2 ↓ Th17↓	(186)
	other Inhibitors	6-bromoindirubin 3’-oxime (BIO)	glycogen synthase kinase 3 (GSK3) STAT3STAT1	xenograft model	prevented	preserved	Th1 ↓Th2 ↓	(187)
other TF Inhibitors	peptide antibiotic	Echinomycin (NSC-13502)	HIF-1α	MHC mismatch model:B6→BALB/c	ameliorated	preserved	Th1 ↓ Th17↓ Tregs↑	(188)
		IT-603	c-Rel	MHC mismatch model:B6→BALB/c	ameliorated	preserved	**-**	(189)
		IT-901	c-Rel	MHC mismatch model:B6→BALB/c	ameliorated	preserved	**-**	(190)
		syntheticretinoid(SR11302)	AP-1	MHC mismatch model:B6→BALB/c	ameliorated	**-**	Th1 ↓ Th17↓ Tregs↑	(191)
		S3I-201	STAT3	MLR (human); human skin graft rejection; xenograft GVHD model; human GVHD	ameliorated	preserved	Th1↓ Th17↓ iTregs↑	(192–194)
	nitrofuran antibiotic	nifuroxazide	STAT3	MHC mismatch model:B6→BALB/c	ameliorated	**-**	Th1↓ Tregs↑	(195)
		bile acid	indirectly RORyt	MHC mismatch model:B6→BALB/c	ameliorated	preserved	Th17 ↓ Treg↑	(196)
		3-OxoLC(bile acid)						(197)

**Table 2:** Summary of clinical trials on Th defining TF inhibitors.

Class	Sub-class	Compound	Target	Clinical trial number	Indication	Co-medication	Effect GVHD	Reference
Kinase inhibitors	JAK/STAT Inhibitors	Ruxolitinib (INCB018424)	JAK1/2	NCT02953678 NCT02913261	Steroid- refractory aGVHD	Corticosteroids, BAT	Ameliorated	(198–200)
Kinase inhibitors	JAK/STAT Inhibitors	Itacitinib (INCB039110)	JAK1	NCT02614612	Steroid-naïve & steroid-refractory GVHD	Corticosteroids	ameliorated	(201)
Kinase inhibitors	JAK/STAT Inhibitors	Pacritinib	JAK2	NCT02891603	aGVHD	Rapamycin (Sirolismus), Tacrolismus	ameliorated	(166)

BAT, best available therapy.

### Epigenetic Modulators

Epigenetic modulation of transcription is a promising approach to indirectly inhibit TF expression. The acetylation of histones, regulated by histone acetyl transferases (HATs) and histone deacetylases (HDACs), is an epigenetic mark, which influences chromatin structure and ultimately gene expression. The use of HDACs and HDAC-inhibitors (HDACi) can modulate this balance and subsequently alter gene expression.

Valproic acid (VPA), a HDACi of the short-chain fatty acid category, was shown to indirectly decrease STAT5 phosphorylation and dampen T-bet expression in NK cells (202). In a mouse model, the administration of VPA attenuated aGVHD by downregulation of Th1 and Th17 cells (171). This effect was associated with a direct inhibition of Akt (171), a kinase which promotes Th1, Th17 and Tfh but inhibits Treg development by activation of mTOR which in turn induces T-bet, RORγt and HIF1α and inhibits FOXO1-dependent FoxP3 transcription (203–205). Importantly, the GVL-effect was preserved during VAP therapy.

Another HDACi, which showed promising effects on GVHD in preclinical models is Ex-527, a Sirtuin-1 (Sirt-1) inhibitor. Sirt-1 represses AP-1, Smad3 and FOXO-transcription factors which regulate pTreg differentiation *via* the CNS1 regulatory element (206–209) and was identified as a direct negative regulator of FoxP3 (210). Pre-clinical experiments in a murine GVHD mode showed that Sirt1^-/-^ T cells were impaired in inducing aGVHD and showed an enhanced pTreg differentiation in which FoxP3 stability was increased. Ex-527 administration induced comparable effects while preserving GVL effects (172). Stabilization of FoxP3 expression by Ex-527 had already been reported earlier and associated to increased Treg suppressive function (210, 211). Another Sirt-1 inhibitor, Sirtinol, was found to decrease RORγt and IL-17A expression in CD4 T cells *in vitro* and to screw Th17/Treg differentiation towards Tregs, leading to a prolonging allograft survival in a mouse transplantation model (212). However, the effects of Sirnotol in GVHD were not reported yet.

Givinostat (ITF2357), a HDACi of the hydroxamic acid category, was also reported to suppress Th17 polarization and enhance FoxP3 expression and hence Treg differentiation *via* decreased STAT3 phosphorylation and RORγt expression downstream of IL-6R signaling. Administration of Givinostat inhibited experimental colitis development by skewing the Th17/Treg balance in the lamina propria (213) and reduced release of inflammatory IFN-γ and TNF-α in systemic inflammation (214). Virinostat (SAHA), another hydroxamic acid HDACi, inhibits STAT3 and also STAT1 phosphorylation, and was shown to attenuate GVHD and inhibit proinflammatory cytokine production during the initiation phase of GVHD (173). Additionally, blocking of STAT3 by both Givinostat and Virinostat, was shown to enhance indoleamine 2,3-dioxygenase (IDO) expression in APCs which suppresses APC allo-stimulatory functions and reduced GVHD in a murine allogeneic BM-transplantation model (215). Hence, Givinostat and Virinostat attenuate GVHD *via* multiple mechanisms, targeting inflammatory cytokine release, antigen presentation and T cell differentiation (216).

Romidepsin (Istodax), a cyclic peptide class HDACi, was shown to effectively suppress allo-responses in a mixed lymphocyte reaction (MLR) (174). Recently, Romidepsin was also shown to inhibit the activation of STAT1 and STAT3 *via* induction of suppressor of cytokine signaling 1 (SOCS1) (175). However, its effect in GVHD has not been fully assessed yet as a first study in patients was terminated due to slow accrual (clinical trail.gov, NCT02203578).

Overall, epigenetic modulators like the describes HDACi, were shown to efficiently inhibit GVHD by altering Th polarization *via* TF modulation. The success of HDACi in preventing GVHD is also displayed by multiple clinical studies validating their beneficial effect often in combinatory therapy in patients [reviewed by Xu et al., (217)].

### Kinase Inhibitors

Another indirect way to target TFs is the inhibition of Kinases, which catalyze the transfer of activating phosphate from ATP to substrates withing signaling pathways. Hence, kinase inhibitors can indirectly block activation of the respective kinase substate like TFs and hence modulate transcription.

The most prominent examples in GVHD therapy are Janus-Kinase (JAK) inhibitors. These inhibitors block JAK/STAT signaling pathways, which have a crucial function of transmitting cytokine-receptor signals intracellularly. Early expression profiling studies and the detection of activated STAT1 and STAT3 in GHVD target organs and alloreactive donor T cells already indicated a link between GVHD and cytokine signaling through the JAK/STAT pathway (218–220).

Subsequent experiments, disrupting JAK/STAT1 signaling by the use of T cells lacking STAT1, a Th1 specific TF responding to IFN-γ Receptor (IFNγR) signaling, reported ameliorated GVHD outcomes in a minor antigen-mismatched and fully-MHC mismatched GVHD model (155). Shortly after, Ruxolitinib (INCB018424), a bioavailable JAK1/2 inhibitor, was reported to have similar mitigating effects on GVHD as IFNγR^-/-^ T cells while the GvL effect was preserved (176, 177, 221, 222). Further mechanistical analyses revealed, that Ruxolitinib ameliorates GVHD by disrupting Th1 and Th17 differentiation but promoting Treg differentiation *via* indirect STAT1 and STAT3 inhibition (223). Overall, these pre-clinical data suggested Ruxolitinib as a promising candidate for GVHD treatment, which indeed has shown remarkable results in the application for steroid refractory GVHD in various clinical studies (224).

Besides Ruxolitinib inhibiting JAK1 and JAK2 simultaneously, selective JAK1, JAK2 and JAK3 inhibitors have also been investigated as potent treatment options in GVHD. The JAK3 inhibitor Tofacitinib (CP-690550) was reported to ameliorate GVHD *in vivo* and *in vitro* by selectively inhibiting Th1 differentiation but not Th17 polarization or CD4 T cell proliferation (183). Itacitinib (INCB039110), a selective JAK1 inhibitor, disrupts the JAK1/STAT3 signaling pathway and was shown to improve GVHD outcomes and survival in various mouse models, partially by reduction of CD4 and CD8 T cell numbers in the inflamed colon tissue, indicating a loss of Th17 phenotype (178–180). Itacitinib also showed promising efficiencies in the treatment of steroid-naïve and steroid-refractory GVHD in a first clinical study (201). Selective inhibition of the JAK2/STAT3 axis, an IFN-γ, IL-6 and IL-23 receptor signaling response element, by Pacritinib (SB1518) was also shown to significantly reduce GVHD in murine models (181, 225). Similar to the effects of the JAK/STAT3 inhibitor Fedratinib in early MLR experiments; Pacritinib, led to impaired expansion of Th1 and Th17 cells while Treg and Th2 responses were sustained (181, 182). A recent study also reported a successful combinatory therapy of acute GVHD with Pacritinib the STAT3 inhibitor S31-201 and the mTOR inhibitor Rapamycin in a xenogeneic mouse model and with Rapamycin and the calcineurin inhibitor Tacrolismus in patients (166).

Despite the advanced clinical validation of JAK/STAT inhibitors in GVHD [reviewed by Assal and Mapara, (224)], few other agents of the Kinase-inhibitor group have also shown beneficial effect on GVHD in pre-clinical studies. Inhibition of the glycogen synthase kinase 3 (GSK3) by the small molecule 6-bromoindirubin 3’-oxime (BIO), prevented mice from lethal GVHD in a xenogeneic model by STAT1/3 suppression and subsequent decrease of Th1 effector cytokines (187). Recent studies suggested the IL-2 inducible kinase (ITK) inhibitor ONO-7790500 as another potent therapeutic in GVHD, as administration inhibited Th1, Th2 and Th17 differentiation, inflammatory cytokine production and alloreactive T cell proliferation and significantly delayed GVHD onset and mortality (186). An earlier study with ITK^-/-^ donor T cells in an allo-HSCT mouse model has already reported comparable beneficial effects on GVHD and observed reduced expression of IRF4, JAK1, JAK2, and STAT3 as well as phosphorylated forms of JAK1, JAK2 and STAT3 if ITK was absent in T cells, which might explain impaired differentiation capacities observed in the ITK inhibitor study (226). Rho-Kinase (ROCK) inhibitors represent a further group evaluated in pre-clinical GVHD settings. While Fausidil, a small molecule inhibiting ROCK1 and ROCK2 only had moderate ameliorating effects on GVHD-associated colitis (184), the ROCK2 inhibitor Belomosudil (KD025), which shifts the Th17/Treg balance towards homeostasis *via* an STAT3/STAT5-dependent mechanism, efficiently ameliorated chronic GVHD in multiple models and first clinical studies (185, 227). However, the effect of Belomosudil on aGVHD remains to be determined.

### Other Direct and Indirect Transcription Factor Inhibitors

Besides epigenetic regulators and kinase inhibitors other small molecules targeting TFs in a direct or indirect manner have been assessed in pre-clinical GVHD models in the last decade.

As already implicated by the successful use of JAK/STAT inhibitors, the repression or STAT3, an important activator of RORγt during Th17 differentiation, was investigated as potent strategy to prevent severe GVHD. Betts at al. reported, that the small molecule S3I-201 efficiently inhibits STAT3 expression, leading to suppressed proliferation of allo-sensitized T cells and impaired Th17 differentiation while iTreg polarization was enhanced. Mechanistically, the group uncovered that S3I-201 polarized the phosphorylation of STAT5 over STAT3 and led to activation of FoxP3 in iTregs (192). Hence, S3I-201 shifts the Th17/Treg balance towards regulatory T cells, as already reported for other STAT3 inhibitors in this review earlier. A later study of the group connected increased pSTAT3 and RORγt levels with severe aGVHD. They found that RORγt suppression was enhanced by combinatory treatment with Rapamycin and S3I-201, which abrogated the proliferation of Rapamycin-resistant T cells upon allo-sensitization in a MLR model (193). Additionally, they reported successful prevention of acute GVHD in a xenogeneic mouse model, using a combinatory treatment with S31-201, the JAK2 inhibitor Pacritinib and Rapamycin in a recently published study, as referred to earlier (166). Moreover, S3I-201 treated iTregs were found to efficiently reduce skin graft rejection and GVHD in a xenograft mouse model by reducing Th1- and Th17-mediated allorectivity, while preserving the GVL effect (194). Similarly, the STAT3 inhibitor nifuroxazide also attenuated GVHD symptoms in skin, liver and GI-tract and efficiently delayed aGVHD-associated lethality (195). Blocking of the TF AP-1 by the synthetic retinoid SR11302 also inhibited Th1/Th17 proliferation and enhanced Treg expansion by indirectly pSTAT3 blockage and STAT5 dependent FoxP3 expression, leading to diminished GVHD-associated pathology and lethality (191). Another study, which investigated the effect of GRIM19 overexpressing donor BM and T cells in GVHD, also found decreased disease-severity, Th17 polarization, and alloreactive activation due to diminished STAT3 expression. Comparable to the effect of other STAT3 inhibitors, GRIM19 overexpression also led to enhanced STAT5 expression and Treg differentiation suggesting GRIM19 induction as another potent strategy for STAT3 inhibition in the future (228).

Alongside STAT3, the inhibition of other Th1 and Th17-differentiation inducing TFs was shown to efficiently ameliorate GVHD. Inhibition of HIF1α, a key TF in Th17/Treg reciprocal differentiation, by Echinomycin (NSC-13502) was shown to efficiently attenuate GVHD and preserve anti-leukemic activity by inducing Treg expansion while diminishing Th17 responses (229). The TF c-Rel plays a role in differentiation of Th1, Th17 and Treg cells. Studies on the c-Rel inhibitor IT-603 showed ameliorating effects on GVHD, mediated through reduced alloreactivity, defective gut homing and impaired negative feedback on IL-2 production by effector T cells leading to an expansion of regulatory T cells. The attenuating effects on GVHD were additionally accompanied by a preserved graft-versus-tumor (GVT) effect and promising effects against lymphomas (189, 190). Bile acid synthesized form cholesterol, called 3-oxoLC was discovered as an inhibitory ligand of the RORγt. It efficiently altered Th17/Treg polarization towards regulatory T cells in the lamina propria suggesting a beneficial effect of bile acid metabolites in controlling intestinal-microbiome tolerance but also immune responses in GI-associated GVHD (197). Indeed, a shortly later published study reported, that the bile acid pool was reduced in patients with GVHD, and that application of bile acids reduced GVHD in several transplantation mouse models but was rather associated to alterations in antigen presentation that in Th17 differentiation (196). However, these studies suggest bile acids as potent immune modulators in the GI-tract during GVHD, partially acting through Th-subset determining TF inhibition.

## Conclusion

Summarized, these data show that specific targeting of Th cell-differentiation involved transcription factors might represent a potent therapeutic strategy to prevent or ameliorate GVHD in addition to standard of care medication. However, most of the presented therapeutics have only been assessed in pre-clinical models yet and beneficial effects for patients remain to be proven. In addition, the immune modulatory effect of the presented therapeutic strategies may lead to a higher susceptibility for infections. This includes the re-activation of latent viral infections [e.g. cytomegalovirus (CMV)] but also the predisposition for newly acquired infections due to major immune suppression of especially Th1 T cells but also other immune cell populations required for viral clearance. First clinical trials with the HDACi Vorinostat and Panobinostat in GVHD patients did not show an augmentation for risk of infections while Romidepsin treated patients with T cell lymphoma more often experiences infections (230–232). Studies on the JAK1/JAK2 inhibitor Ruxolitinib also reported an increased susceptibility for viral re-activation of Hepatitis-B and varicella zoster virus in treated patients with myeloproliferative neoplasm and polycythemia vera, but also a modestly higher incidence of infection and reactivated CMV infection in patients with steroid-refractory GVHD (198, 233, 234). However, first line and second line therapies in GVHD also harbor the risk of viral re-activation and overall significant improvement in efficacy outcomes by more target specific TF inhibitors probably weights more than a moderate elevated risk for infection (198, 235). Additionally, the above-mentioned examples from clinical trials show that the risk of an enhanced susceptibility towards infections under TF inhibitor treatment is highly dependent on the drug target and specificity so that these more specific TF inhibitors might exhibit superior protection from infections that other commonly used therapeutics.

Together, given the promising results of some TF-modulators in clinical studies, we expect a fundamental contribution of TF-inhibitors to improve GVHD therapy in the future.

## Author Contributions

JC and EU wrote the manuscript and approved the submitted version.

## Funding

The laboratory of EU has been supported by the Frankfurt Cancer Institute FCI/DKTK (to EU), by the German Research Foundation DFG (CRC 1292, UL316/5-1), by the German Cancer Aid, the “Alfred & Angelika Gutermuth-Stiftung” and by “Menschen für Kinder e.V.”

## Conflict of Interest

The authors declare that the research was conducted in the absence of any commercial or financial relationships that could be construed as a potential conflict of interest.

## Publisher’s Note

All claims expressed in this article are solely those of the authors and do not necessarily represent those of their affiliated organizations, or those of the publisher, the editors and the reviewers. Any product that may be evaluated in this article, or claim that may be made by its manufacturer, is not guaranteed or endorsed by the publisher.
